# Mapping Social and Physical Frailty in the Peruvian Amazon: Associated Factors Among Older Adults

**DOI:** 10.3390/healthcare14121684

**Published:** 2026-06-12

**Authors:** Fernando M. Runzer-Colmenares, Walter Mendoza, Nelson Luis Cahuapaza-Gutierrez, Kiara Camacho-Caballero, Jose F. Parodi

**Affiliations:** 1 CHANGE Research Working Group, Universidad Científica del Sur, Lima 15067, Peru; 100065659@cientifica.edu.pe (N.L.C.-G.);; 2 Facultad de Ciencias de la Salud, Universidad Científica del Sur, Lima 15067, Peru; 3 Centro de Investigación del Envejecimiento, Facultad de Medicina Humana, Universidad de San Martín de Porres, Lima 15067, Peru

**Keywords:** social frailty, frailty, physical frailty, Peruvian Amazon, geriatric medicine

## Abstract

**Background/Objectives**: To analyze the determinants of frailty among older adults residing in the Peruvian Amazon, focusing on four complementary frailty-related outcomes. **Methods**: A cross-sectional study was conducted using secondary data from the Amazon frail study. Four multivariable Poisson regression models were specified. Associations were estimated using prevalence ratios (PRs) with their corresponding 95% confidence intervals (95% CIs), employing robust variance estimation to account for potential heteroskedasticity. **Results**: The sample included 429 older adults (≥60 years), predominantly female (64.0%), with most participants aged 60–79 years (85.8%). The prevalence of the main variables of interest was: dynapenia (51.5%), low physical performance (32.2%), social frailty (66.4%), and frailty according to the Fried phenotype (26.3%). In adjusted models, altered muscle mass showed the strongest association with dynapenia (PR = 2.08, 95%CI = 1.74–2.50). Likewise, functional dependence and social frailty were significantly associated with low physical performance (PR = 1.65 and PR = 1.57, respectively). A higher potential support relationship (upper tertile) was associated with a lower prevalence of social frailty (PR = 0.56, 95%CI = 0.41–0.88), whereas low physical performance was strongly associated with frailty defined by the Fried phenotype (PR = 3.71, 95%CI = 2.60–5.34). **Conclusions**: Frailty among older adults in the Peruvian Amazon is highly prevalent and characterized by a distinctly multidimensional nature. It is associated with muscular, functional, cognitive, and social factors that may reflect interrelated vulnerabilities at both the individual and community levels.

## 1. Introduction

In response to the rapidly changing demography of Peru, driven by decreasing fertility and increased survival, aging is increasingly mainstreamed in public debate and policymaking in Peru. The COVID-19 pandemic put them at the very core of concern, with Peru being one of the countries most affected. Nevertheless, approaches to elderly populations usually overlook their heterogeneities and intersectional implications, such as ethnic, cultural, or territorial [1].

In that context, frailty, dynapenia, poor physical performance, and physical performance phenotypes are highly prevalent among older adults and are increasingly recognized as core geriatric syndromes rather than isolated conditions. Frailty is a complex and multidimensional biopsychosocial syndrome [2]. Physical frailty, in turn, is defined as a state of vulnerability characterized by diminished physical reserve, reduced resilience to stressors, increased physiological susceptibility, and a greater propensity for disease [3]. Dynapenia refers to the age-related loss of muscle strength and power, regardless of muscle mass [4]. Impaired physical performance, on the other hand, is a central component of physical frailty and, together with unintentional weight loss, exhaustion, and low levels of physical activity, constitutes one of the key domains used to characterize the frailty phenotype in older adults [5].

In diverse settings, low muscle strength, reduced gait speed and impaired lower extremity performance cluster with advanced age, multimorbidity, malnutrition, physical inactivity, and low educational and socioeconomic status. These syndromes are not benign: They are strongly linked to cognitive impairment and depressive symptoms, loss of independence in basic and instrumental activities of daily living, decline in intrinsic capacity (specifically in the vitality domain), falls and fractures, unplanned hospitalizations, institutionalization, and premature mortality. Taken together, they mark a transition from robust to vulnerable aging and represent a major challenge for health systems that try to support older adults’ autonomy and quality of life.

Evidence from rural contexts suggests that these problems may be even more pronounced outside large urban centers, but remain fragmentary. Studies from rural regions of South America, India, Central America, and several African countries describe high burdens of functional limitation, sarcopenia, and cognitive decline, often concentrated among older women with low schooling and lifelong involvement in physically demanding agricultural work [6]. At the same time, access to primary care is constrained by long travel distances, seasonal river or road conditions, shortages of trained professionals and diagnostic tools, and strong dependence on traditional medicine. In these environments, most frailty and sarcopenia interventions, largely developed and tested in urban or high-income settings, have uncertain feasibility and effectiveness, and social frailty has received comparatively little attention. For the rural Peruvian Amazon in particular, the available data point to marked vulnerabilities but also highlight the major gaps in epidemiological evidence and culturally appropriate care models [7,8,9,10,11,12,13,14].

These observations support the need to approach frailty as a multidimensional construct that integrates physical, cognitive, emotional, and social domains and is shaped by the demographic context in which older adults live, including living and housing conditions, such as living alone or in a companion. Understanding how functional dependence, cognitive impairment, and depressive symptoms interact with community-level demographic indicators such as age structure, potential support ratios, and the demographic dividend can help identify older adults at the highest risk and design person-centered strategies that are realistic for low-resource Amazonian settings. Generating such context-specific evidence is especially relevant for primary care teams in the Amazon basin, who must prioritize limited resources while responding to complex and overlapping needs in aging populations [15,16,17,18,19,20,21,22,23,24].

Against this background, the present study aimed to examine, in older adults from rural communities of the Peruvian Amazon, the associations of functional status, cognitive impairment, depressive symptoms, and community-level demographic indicators with four complementary outcomes related to frailty: dynapenia, physical performance, social frailty, and frailty according to the Fried phenotype.

## 2. Materials and Methods

### 2.1. Study Design and Population

This cross-sectional, observational, and analytical design was based on secondary data from the original Amazon Frail project. The primary study used a non-probabilistic census-type sampling strategy, recruiting over 95% of eligible residents. The Amazon Frail study included community-dwelling adults aged ≥ 60 years from suburban and rural areas in the Peruvian Amazon departments of Loreto, San Martín, and Ucayali. These communities were dispersed, with low population density, usually isolated without remote technological access. Recruitment was conducted during the 2024–2025 period. Data collection was carried out by trained healthcare professionals—physicians with expertise in geriatrics and epidemiology through standardized direct interviews, ensuring the quality, consistency, and validity of the recorded data and measurements.

### 2.2. Population

The sample comprised 429 participants from the Amazon Frail study. Inclusion criteria were age ≥ 60 years and residence in the selected communities. Participants with a diagnosis of severe dementia (ICD-10: F03.C2) and those unable to provide informed consent were excluded; in cases of illiteracy—more common in the Peruvian Amazon and among women—verbal informed consent was obtained in accordance with established ethical procedures.

### 2.3. Variables

For the analysis of physical and social frailty variables among residents of the Peruvian Amazon, the following clinical and functional variables were considered: Physical performance was assessed using the Short Physical Performance Battery (SPPB) with a cutoff of ≤8 points as proposed by the sarcopenia consensus [25]. Dynapenia was defined using grip strength cut-offs from the consensus on sarcopenia. The strength was assessed three times, and the average value was recorded. Fried’s Frailty Phenotype was determined by ≥3 of the following: gait speed < 0.8 m/s [25], low grip strength per consensus criteria [25], self-reported weight loss (Edmonton Frail Scale item) [26], low physical activity (SHARE Frailty Index item) [27]. Social frailty was measured using the Social Frailty Index (SFI), previously translated and validated into Spanish by an expert consensus. The upper tertile is considered positive [28].

Functional dependence was defined as a Barthel Index ≤ 60 [29]. Depressive symptoms were evaluated using the Yesavage scale (3/5 items) [30]. Cognitive impairment was determined by the Pfeiffer test (≥4 errors, adjusted for education) [31]. Muscle mass alteration was calculated using the Skeletal Muscle Mass Index (SMMI) for sex-specific cutoffs: SMMI < 7.26 kg/m^2^ for men and SMMI < 5.45 kg/m^2^ for women [32]. Multimorbidity was defined as ≥2 medical conditions (Coronary artery disease, cerebrovascular accident, chronic pain, hypertension, type 2 diabetes, chronic obstructive pulmonary disease, heart failure, cancer, asthma, osteoarthritis, chronic kidney disease, Dengue, Chagas, Hepatitis B, Leishmaniasis, Malaria, syphilis, others) [33]. The age dependency ratio tertiles and the potential support ratio tertiles were derived from official Peruvian census data at the community level, categorized into tertiles for analysis (lower/medium vs. upper tertile) (https://infopoblacion.mimp.gob.pe/pyd (accessed on 1 May 2026)). Age and sex were also included as covariates.

### 2.4. Statistical Analysis

Descriptive analysis was performed using frequencies and percentages for categorical variables. Four Poisson regression models were constructed with the following dependent variables: Fried frailty, dynapenia, social frailty, and physical performance (SPPB). All study covariates were included except those causing over-adjustment. Age was excluded from the social frailty model because it constitutes one of the components used to calculate the SFI. Likewise, sex was not included in the dynapenia model, as the cutoff values used to define dynapenia are sex-specific. Finally, dynapenia was not included in the model corresponding to the Fried frailty phenotype because it constitutes one of the criteria that comprise this definition of frailty.

Community-level demographic indicators were categorized into tertiles according to their distribution and entered the regression models as categorical variables. Because the frailty-related outcomes evaluated in this study (dynapenia, impaired physical performance, social frailty, and Fried frailty phenotype) share certain conceptual and biological domains, they were analyzed as separate outcomes using distinct multivariable models.

Model assumptions of linearity, Poisson distribution, and independence were verified; multicollinearity was assessed using variance inflation factors (VIF), considering VIF ≥ 5 as the cutoff. Prevalence ratios (PR) and 95% confidence intervals were calculated with robust variance estimation to account for potential heteroskedasticity. Pseudo R^2^ values were calculated for each model to evaluate explanatory power and goodness of fit. These metrics provide insight into the proportion of outcome variance explained by the covariate set, facilitating model comparison across frailty domains and evaluation of multivariable adjustment adequacy in this Peruvian Amazon older adult population [34].

Post hoc power calculations were performed for the four Poisson models assuming α = 0.05 and a minimum detectable PR of 1.20. Power ranged from 88.5% (Fried frailty phenotype, 26.3% prevalence) to 83.9% (social frailty, 66.4% prevalence), with an overall minimum power of 88.5% across outcomes. For Fried frailty, the minimum detectable effect at 80% power was PR = 1.31, indicating adequate sensitivity for moderate-to-large associations in this sample size. Analyses were conducted using Stata 19 SE software (StataCorp, College Station, TX, USA).

### 2.5. Ethical Considerations

Data collection procedures strictly adhered to ethical standards, ensuring the integrity, confidentiality, and anonymity of all participants. The recruitment was carried out after obtaining informed consent from each participant, accompanied by a family member. The study protocol was reviewed and approved by the Research and Ethics Committees of the Universidad Científica del Sur and Universidad de San Martin de Porres. The database used for the analysis is fully anonymous. This study was funded by the Vicerrectorado de Investigación de la Universidad Científica del Sur through the Fondo Semilla 2023 and by the Instituto de Investigación de la Facultad de Medicina of Universidad de San Martín de Porres.

## 3. Results

### 3.1. Sample Characteristics

The sample included 429 older adults (≥60 years). A predominance of females was observed (64.0%), with a female-to-male ratio of 1.77. Most participants were aged 60 to 79 years (85.8%). Regarding clinical and functional conditions, 72.96% had multimorbidity, 20.28% cognitive impairment, 37.38% depressive symptoms, 38.55% reduced muscle mass as measured by bioelectrical impedance analysis (BIA), and 38.55% functional dependence.

With respect to the main study variables, the prevalence of dynapenia was 51.52% (n = 221), low physical performance measured using the SPPB was observed in 32.17% (n = 138), social frailty in 66.43% (n = 285), and Fried’s Frailty Phenotype in 26.34% (n = 113). Detailed characteristics of the analyzed sample are presented in Table 1.

### 3.2. Multivariate Associated Factors

Table 2 presents the Poisson regression analyses used to evaluate factors associated with physical and social frailty. Four multivariable models were specified. Model 1 included dynapenia as the primary exposure variable, adjusted for the full set of covariates. Model 2 considered physical frailty as the outcome of interest, with adjustment for the same covariates. Model 3 evaluated social frailty as the dependent variable, incorporating all covariates in the adjustment. Finally, Model 4 included frailty defined according to the Fried phenotype, likewise adjusted for the complete set of variables.

In adjusted Poisson models, alteration in muscle mass (BIA) showed the strongest association with dynapenia (PR = 2.08, 95%CI = 1.74–2.50), while functional dependence and social frailty were associated with physical performance (PRs = 1.65 and 1.57, respectively); potential support relationship (upper tertile) was associated with a lower prevalence of social frailty (PR = 0.56, 95%CI = 0.41–0.88), and physical performance strongly associated with Fried frailty (PR = 3.71, 95%CI = 2.60–5.34) (Table 2). McFadden’s Pseudo R^2^ values indicated model fit: 0.178 (dynapenia), 0.183 (Impaired physical performance), 0.137 (social frailty), and 0.219 (Fried phenotype), highlighting the interconnection between physical, muscular, and social domains in Peruvian aging.

## 4. Discussion

This cross-sectional study of 429 community-dwelling older adults from urban and rural areas shows that frailty is highly prevalent and clearly multidimensional in this setting. Social frailty was the most frequent condition, affecting roughly two-thirds of participants, followed by dynapenia, impaired physical performance (SPPB), and Fried’s frailty phenotype. Across domains, reduced muscle mass, impaired physical performance, and cognitive impairment emerged as constant frailty correlations, while functional dependence was especially linked to physical and social frailty. In particular, higher potential support ratios at the community level appeared to buffer social frailty, and physical performance strongly predicted the Fried phenotype, underscoring how muscular, functional, and social dimensions interact to shape vulnerability in advanced age. Together, these findings highlight the transition from robust to vulnerable aging as a process in which structural demographic constraints and individual deficits converge in the Peruvian Amazon [35,36].

Frailty in this population cannot be reduced to a purely physical construct, even when operationalized through performance-based tests or the Fried phenotype. Although the Fried model captures homeostenosis and a physical vulnerability state characterized by low gait speed, poor strength, weight loss, and low activity, it does not fully encompass cognitive decline, depressed mood, or social disconnection, which have been conceptualized as cognitive frailty, depressed frail phenotype, and social frailty, respectively. Cognitive frailty, defined by the coexistence of physical performance and cognitive impairment, has been associated with accelerated functional decline, disability, and mortality in Latin American and Asian cohorts [20]. Similarly, the depressed frail phenotype and social frailty identify subgroups at particularly high risk of falls, institutionalization, and poor quality of life [37]. Deficit accumulation indices capture this multidimensionality by integrating comorbidities, symptoms, and social factors, while the current study adopted a more domain-specific approach, examining parallel phenotypes that together approximate a multidimensional view of frailty [8,21,38,39,40].

The pattern of associations found in this Amazonian cohort aligns with and extends previous evidence from rural Peru, Brazil, and other low-resource settings. Previous studies have documented strong links between low muscle strength, poor mobility, and malnutrition with dynapenia and physical performance, as well as their role in predicting hospitalizations, disability, and mortality. The robust association observed here between altered muscle mass and dynapenia, and between impaired physical performance and Fried- and SPPB-defined frailty, is consistent with this literature and emphasizes the centrality of the muscular–functional axis in frailty pathways. Similarly, the observation that cognitive impairment is related to dynapenia, physical performance, and social frailty resonates with reports that cognitive frailty triples the risk and accelerates loss of independence in older adults. The co-occurrence of social frailty with functional dependence and dynapenia mirrors Colombian and Asian findings in which living alone, poor social support, and poverty amplify physical and cognitive vulnerabilities [41,42,43,44,45].

These multidimensional patterns also point to several opportunities for intervention framed within the intrinsic capacity paradigm. The consistent links between diminished muscle mass, dynapenia, and physical performance support the implementation of low-cost strength and balance training, combined with nutritional support, as the cornerstones of person-centered care in rural primary care teams. At the same time, the associations of cognitive impairment and social frailty with physical vulnerability suggest that screening for cognition, mood, and social isolation should accompany physical assessments, using tools such as RUDAS and brief social frailty instruments that have been applied in similar contexts. Because higher potential support ratios were protective against social frailty, community and family-based strategies that reinforce intergenerational support, social participation, and locally meaningful roles for older adults can enhance intrinsic capacity beyond what purely biomedical interventions can achieve. In practice, this advocates for integrated community-embedded programs that jointly address physical performance, nutrition, cognition, mood, and social connection. So, understanding frailty as a pre-disability state, the Fried phenotype offers a powerful clinical construct because it translates homeostenosis into physical domains that mirror multisystem decline. Within this framework, slow gait and muscle weakness appear to concentrate a substantial portion of neurodegenerative risk even before overt cognitive impairment, suggesting that longitudinal monitoring of physical and muscular performance should occupy a central place in the identification of frailty and dementia risk, rather than being treated as a mere complement to cognitive testing. Recent work has proposed physical vitality (muscle health plus nutrition) as an underlying substrate rather than an isolated domain, introducing a pre-frailty stage within Fried-based models that aligns closely with the intrinsic capacity framework. This perspective reinforces the leading role of the physical and nutritional factors identified in the present study as early drivers in the trajectory toward frailty and cognitive decline [46,47,48,49,50].

Educational attainment is associated with higher health literacy, which facilitates the appropriate use of healthcare services, greater engagement with social programs and preventive activities, and better adherence to medical treatments. Consequently, higher educational levels have been associated, as reported in previous studies, with a lower prevalence of physical and social frailty markers, as well as cognitive impairment [51].

Beyond its biological and functional dimensions, frailty can also be understood as an indicator of care complexity. A recent multicenter study involving older adults hospitalized with heart failure evaluated frailty and nursing care complexity at hospital admission. The findings showed that frailty was associated with nursing care complexity, and that both frailty and nursing care complexity were independently associated with length of hospital stay. These results suggest that the combined assessment of frailty and nursing care complexity at admission may contribute to the early identification of heart failure patients at risk for prolonged hospitalization [52].

Nursing care complexity has been conceptualized as a multidimensional construct that reflects both patients’ care needs and the intensity of the care required. However, its operationalization has varied considerably across studies, with the use of different clinical and administrative indicators, highlighting the absence of a universally accepted definition [52]. In this context, several studies have employed nursing-related indicators, such as the number of nursing diagnoses documented at hospital admission, as a pragmatic approach to quantifying care complexity and patient care needs in real-world clinical settings [53,54]. Recent evidence suggests that frailty and nursing care complexity represent complementary dimensions that, when considered together, can identify patient profiles with greater care needs, increased healthcare resource utilization, and poorer clinical outcomes. This perspective expands the traditional concept of frailty beyond biological vulnerability, linking it directly to care workload and the organizational challenges faced by healthcare systems [52].

Recent evidence further suggests that frailty and nursing care complexity jointly identify clinically relevant patient profiles associated with adverse health outcomes and increased healthcare service utilization. From this perspective, the multidimensional patterns observed in the present study may have implications not only for individual risk assessment but also for healthcare planning and resource allocation in underserved Amazonian settings.

Several limitations must be considered when interpreting these findings. First, the cross-sectional design excludes the establishment of temporal or causal relationships between frailty domains, cognitive impairment, depressive symptoms, and demographic factors, raising the possibility of bidirectional or reverse causation. Second, although coverage of eligible residents was high, the nonprobabilistic census-type sampling and the restriction to selected communities may limit generalizability to other Amazonian settings. Third, some tools, particularly the Social Frailty Index and the Fried phenotype, have not been fully validated for the Peruvian Amazon, and misclassification cannot be ruled out. Residual confounding is also possible, as not all environmental and healthcare access variables could be included without overadjustment. On the contrary, the strengths of the study include the simultaneous assessment of four outcomes related to frailty, the incorporation of demographic indicators, and the use of robust Poisson regression with high statistical power to explore multidomain associations in an understudied population. Finally, as this was a secondary analysis of an existing database, the study was limited to the variables originally collected, which prevented the inclusion of potentially relevant factors that were not available in the dataset. In addition, the original database did not include community-level weighting procedures, and intracommunity correlation was not explicitly modeled. Therefore, some degree of residual clustering may be present, which should be considered when interpreting the findings.

Future research should prioritize longitudinal designs to clarify causal pathways, validate culturally adapted frailty and social frailty instruments, and test integrated community-based interventions that combine exercise, nutritional support, cognitive stimulation, psychosocial components, and strategies to strengthen family and community support networks. Such efforts are essential if health systems in the Amazon basin are to respond effectively to rapid population aging while preserving autonomy, function, and quality of life among older adults living in rural and resource-limited settings [45,46].

## 5. Conclusions

This study shows that frailty in older adults from urban and rural Peruvian Amazonian communities is common and deeply multidimensional, and is associated with intertwined muscular, functional, cognitive, and social factors that may reflect individual and community-level vulnerabilities. The findings support moving beyond a purely physical concept of frailty towards an approach anchored in intrinsic capacity, in which early identification of dynapenia, impaired physical performance, cognitive impairment, depressive symptoms, and social frailty may help inform personalized, person-centered interventions.

## Figures and Tables

**Table 1:** Descriptive analysis of the variables (n = 429).

Variable	n (%) ^a^
**Age**	
60–79 years	368 (85.78)
≥80 years	61 (14.22)
**Sex**	
Female	274 (64.02)
Male	154 (35.98)
**Old-age dependency**	
Lower/medium tertile (lower burden)	382 (89.46)
Upper tertile (higher burden)	45 (10.54)
**Potential support**	
Lower/medium tertile (higher risk)	379 (88.76)
Upper tertile (lower risk)	48 (11.24)
**Multimorbidity**	
No	116 (27.04)
Yes	313 (72.96)
**Cognitive impairment**	
No	342 (79.72)
Yes	87 (20.28)
**Depressive symptoms**	
No	268 (62.62)
Yes	160 (37.38)
**Muscle mass alteration (BIA)**	
No	263 (61.45)
Yes	165 (38.55)
**Functional dependence**	
No	286 (67.14)
Yes	140 (32.86)
**Physical performance**	
Normal	291 (67.83)
Impaired	138 (32.17)
**Social frailty**	
No	144 (33.57)
Yes	285 (66.43)
**Dynapenia**	
No	208 (48.48)
Yes	221 (51.52)
**Fried’s Frailty Phenotype**	
Normal	316 (73.66)
Frail	113 (26.34)

^a^ frequencies and percentages. BIA: bioelectrical impedance analysis.

**Table 2:** Multivariate Poisson regression models to determine factors associated with frailty, frailty components, and social frailty. (n = 429).

Variable	Model 1	Model 2	Model 3	Model 4
**Old-age dependency**				
Upper tertile (higher burden)	1.11 (0.89–1.43)	1.17 (0.77–1.73)	0.83 (0.67–1.05)	1.02 (0.66–1.54)
**Potential support relationship**				
Upper tertile (lower risk)	0.87 (0.64–1.18)	0.65 (0.37–1.15)	0.56 (0.41–0.88)	0.83 (0.49–1.53)
Cognitive impairment (Yes)	**1.35 (1.13–1.62)**	**1.51 (1.16–2.07)**	**1.14 (1.06–1.30)**	1.02 (0.73–1.46)
Depressive symptoms (Yes)	1.07 (0.89–1.26)	1.19 (0.91–1.58)	1.11 (0.99–1.26)	1.06 (0.77–1.46)
Muscle mass alteration (Yes)	**2.08 (1.74–2.50)**	0.85 (0.64–1.12)	**1.25 (1.10–1.44)**	1.51 (1.16–2.04)
Functional dependence (Yes)	**1.26 (1.06–1.54)**	**1.65 (1.25–2.19)**	**1.33 (1.18–1.50)**	0.94 (0.69–1.28)
Physical performance (Impaired)	**1.22 (1.06–1.50)**	—	1.16 (1.00–1.29)	**3.71 (2.60–5.34)**
Social frailty (Yes)	**1.32 (1.02–1.76)**	**1.57 (1.04–2.36)**	—	1.23 (0.82–1.83)
Dynapenia (Yes)	—	**1.61 (1.16–2.20)**	**1.16 (1.02–1.38)**	—

Pseudo R^2^: Model 1 (Dynapenia): 0.178; Model 2 (Impaired physical performance): 0.183; Model 3 (Social frailty): 0.137; Model 4 (Fried frailty): 0.219. Bold values indicate a higher prevalence ratio in the multivariate Poisson regression models assessing factors associated with frailty, frailty components, and social frailty.

## Data Availability

The data presented in this study are available on request from the corresponding author due to the fact that the project has completed data collection; however, final reports have not yet been closed, and therefore the dataset is not currently available for unrestricted access.

## Abbreviations

The following abbreviations are used in this manuscript:

BIA	Bioelectrical impedance analysis
PR	Prevalence ratios
SFI	Social Frailty Index
SMMI	Skeletal Muscle Mass Index
SPPB	Short Physical Performance Battery
VIFs	Variance inflation factors
